# The Graded Fate of Unattended Stimulus Representations in Visuospatial Working Memory

**DOI:** 10.3389/fpsyg.2019.00374

**Published:** 2019-02-26

**Authors:** Muhammet I. Sahan, Edwin S. Dalmaijer, Tom Verguts, Masud Husain, Wim Fias

**Affiliations:** ^1^Department of Experimental Psychology, Ghent University Ghent, Belgium; ^2^Department of Experimental Psychology, University of Oxford Oxford, United Kingdom; ^3^MRC Cognition and Brain Sciences Unit, University of Cambridge Cambridge, United Kingdom

**Keywords:** spatial attention, working memory, memory quality, binding errors, distance effects

## Abstract

As in visual perception, information can be selected for prioritized processing at the expense of unattended representations in visual working memory (VWM). However, what is not clear is whether and how this prioritization degrades the unattended representations. We addressed two hypotheses. First, the representational quality of unattended items could be degraded as a function of the spatial distance to attended information in VWM. Second, the strength with which an item is bound to its location is degraded as a function of the spatial distance to attended information in VWM. To disentangle these possibilities, we designed an experiment in which participants performed a continuous production task in which they memorized a visual array with colored discs, one of which was spatially retro-cued, informing the target location of an impending probe that was to be recalled (Experiment 1). We systematically varied the spatial distance between the cued and probed locations and obtained model-based estimates of the representational quality and binding strengths at varying cue-probe distances. Although the representational quality of the unattended representations remained unaffected by the cue-probe distance, spatially graded binding strengths were observed, as reflected in more spatial confusions at smaller cue-probe distances. These graded binding strengths were further replicated with a model-free approach in a categorical version of the production task in which stimuli and responses consisted of easily discriminable colors (Experiment 2). These results demonstrate that unattended representations are prone to spatial confusions due to spatial degradation of binding strengths in WM, even though they are stored with the same representational quality.

## Introduction

Working memory (WM) is a fundamental cognitive function that enables humans to temporarily retain information in the absence of sustained sensory input from the physical world. Current accounts of WM suggest that the maintenance of internal representations heavily relies on attentional mechanisms that overlap with those engaged in processing perceptual information (Awh and Jonides, 2001; Postle, 2006; Chun, 2011; Kiyonaga and Egner, 2013). Accordingly, a growing body of research has shown that prioritizing information in WM is characterized by the same behavioral patterns and recruits the same neural systems as those that are engaged in attentional selection in physical space (Gazzaley and Nobre, 2012). Similar to prospectively cued locations in physical space, retrospectively cued locations guide the focus of attention to specific locations in mental space, improving memory performance for the cued memory information, but with a cost to uncued memory information held in WM (Souza and Oberauer, 2016). These findings have triggered the important next question how prioritizing the attended item has an influence on the unattended items in WM.

Previous research indicated that the fate of unattended WM representations depends on the spatial distance between the attended and unattended locations (Rerko and Oberauer, 2013; Rerko et al., 2014; Sahan et al., 2015). In our earlier work (Sahan et al., 2015), we studied the spatial distribution of attentional resources by comparing how well non-targets from uncued locations which were presented at the cued locations were rejected as a function of the distance from their original location. For this purpose, we adapted the pre- and retro-cueing paradigms for guiding attention through physical and mental space, respectively (Griffin and Nobre, 2003; Landman et al., 2003). More precisely, participants were first presented with an array of colored disks. Subsequently they had to decide whether a color probe presented at a cued location matched the color at that location in the originally presented stimulus array they were holding in WM. The ability to correctly reject color probes that were drawn from the uncued locations gradually improved with spatial distance to both pre- and retro-cued locations. Similarly, other studies have shown this effect in production tasks (Emrich and Ferber, 2012; Bays, 2016; Oberauer and Lin, 2017; Pratte, 2018). For instance, Rerko et al. (2014) showed that in a recall task version of the retro-cueing paradigm, recall errors at cued locations were also graded by the spatial distance of the non-target memory information. Specifically, they showed that non-targets from nearby locations to the cued locations were more likely to be confused with the target's color than non-target colors from far away.

Although these studies suggest that the spatial distribution of attention in WM follows a gradient similar to spatial gradients in perceptual processing (Downing and Pinker, 1985; Henderson and Macquistan, 1993; Eimer, 1997), the graded nature of unattended items remains unclear. In principle, there are two possibilities. A first possibility is that the representational quality of unattended memory items becomes spatially graded as a function of their spatial distance to the attended location, with neighboring items being more accurately represented than items at a more distant location. This could be the consequence of a mechanism of attentional spillover, in which the graded allocation of attentional resources facilitates information processing at neighboring locations more than at locations further away (Downing and Pinker, 1985; Schmidt et al., 2002). A second possibility is that the binding of unattended memory items to their specific locations in WM is affected (Bays and Husain, 2008). Specifically, it is likely that the binding strength of unattended representations to their locations is weakened to the degree these items are in the vicinity of attended locations, which could be the consequence of spatial imprecisions in WM (Emrich and Ferber, 2012; Bays, 2016; Oberauer and Lin, 2017). The aim of the current study was to investigate two possible consequences of attentional prioritization on memory items that were not selected for further processing in WM, namely the distance-related degradation of the quality and the distance-related degradation of the item-to-location bindings.

Earlier work did not allow to assess both possibilities simultaneously and, therefore, has not led to a conclusive interpretation. Some studies investigated binding as a function of distance, but could not evaluate the possibility of a spatial gradient in the quality of the memory representations. The tasks used in these studies required binary decisions on whether probes matched their samples (Rerko and Oberauer, 2013; Sahan et al., 2015) or used tasks that required a selection from a limited set of categorical memory alternatives (Rerko et al., 2014). These tasks do not allow to judge the quality of the memory contents. Another type of task, namely the continuous production task, is better suited to reveal the distance related changes in the quality of memory representations. These tasks require a precise reproduction of memory contents from a continuous scale (e.g., by selecting a position on a color wheel displaying the color spectrum) and thus have the advantage of not only revealing whether an item is remembered or not but also providing a continuous measure of the representational quality defined as the precision with which these items are maintained (Wilken and Ma, 2004; Ma et al., 2014). Using such continuous production tasks, it has been shown that the quality of WM representations degrades with larger set sizes and that it is larger for cued information at the expense of uncued information (Gorgoraptis et al., 2011; Pertzov et al., 2013). What has not been examined yet is whether the quality of the memory representation is also subjected to an attentional gradient (Rerko and Oberauer, 2013; Rerko et al., 2014; Sahan et al., 2015). In order to address this question, we tested whether the representational quality of uncued WM information is differentially graded as a function of location with respect to the attentional focus. For this purpose, we systematically varied the spatial distance between the initially retro-cued and eventually probed locations. Specifically, participants were required to precisely reproduce the color of a probed target at a location that could appear at different distances from the cued location (cue-probe distance). In this way we could assess the fate of uncued WM representations in terms of how their quality is influenced -graded or not- by the spatial distance between the cued and probed location. We predicted that the quality of unattended representations would gradually decrease with increasing cue-probe distance if attentional resources spill over to neighboring locations.

The second possibility, next to the graded representational quality, is that the strength of the binding of the unattended stimuli to their location is spatially graded. Spatial locations that are close to each other are similar in terms of their feature space giving rise to spatial imprecisions (Oberauer and Lin, 2017; Schneegans and Bays, 2017). Spatially graded binding errors may arise then as a consequence of spatial imprecisions that are likely to be higher for unattended representations in the vicinity of attended locations. An established way to study these binding errors is to apply theoretical models to the results from a continuous production task. For instance, Zhang and Luck (2008) proposed that WM representations are either normally distributed around the selected memory information (i.e., target), thereby reflecting the correct target responses, or alternatively are uniformly distributed as a manifestation of random guessing in which case errors are made. However, Bays and Husain (2008) pointed out that a considerable amount of errors are not driven by guessing but by erroneously binding memory representations to unattended locations (i.e., non-target). While assessing the overall contribution of the non-targets in recall errors, the role of non-target distances was not taken into account in explaining the emergence of binding errors. Our aim was to investigate the role of non-targets in recall errors, especially with respect to their spatial distance to the target locations, in order to reveal whether binding errors are graded.

In a recent study, Oberauer and Lin (2017) proposed the interference model of WM which suggested that binding errors arise from competition between neighboring WM representations. The model included a spatial gradient parameter weighting the contribution of non-target representations to recall performance. It was able to fit the data of continuous production tasks, hence being suggestive of the validity of the idea of a gradient in binding accuracy. Based on this spatial gradient parameter defined by Oberauer and Lin (2017), we hypothesized that the non-target representations near the target location would be more prone to misbinding compared to non-target representation further away.

Our approach differs from Oberauer and Lin (2017), in that our model does not assume an *a priori* spatially graded parametric function. Instead, we (non-parametrically) calculate the binding errors of the non-target colors from the different locations and verify whether or not they show a spatially graded pattern (i.e., non-target distance effect). For this purpose, we fitted an adapted version of the mixture model proposed by Bays et al. (2009) to our data. The original model decomposed memory performance into target responses, random guesses and binding errors. While these binding errors did not take into account the spatial distance of the non-targets, we further decomposed the binding errors as a function of the spatial distance of non-target locations relative to the target location. In this way we can directly assess the fate of non-target representations and their spontaneous contribution to recall performance with respect to their distance to the probed location without parametrically restricting the spatial distance of the unattended representations in our model. Furthermore, our design also allowed us to investigate whether these binding errors -graded or not- were further modulated in the context of retro-cues. We hypothesized that if spatial imprecisions are higher for unattended representations in the vicinity of attended representations, then shifting attention to those unattended locations would result in even a higher likelihood of binding errors. For this purpose, we varied the spatial distance between cued and probed locations in the same experiment as we studied representational quality and tested whether the binding errors were spatially modulated at each cue-target distance condition (i.e., cue-probe distance effect).

In sum, we aimed to understand the graded fate of unattended items in visual WM after another item has been selected for attentional processing. A model-based approach was adopted that enabled us to tease apart two possible consequences of attentionally selecting a target on the unattended stimuli, namely the distance-related degradation of the quality of the unattended stimuli and the distance-related likelihood of the unattended items to be erroneously bound to the location of the target stimulus.

## Experiment 1

A continuous production task requiring participants to reproduce colors from a full color spectrum was administered in order to gain direct insight into the consequences of attentional selection in WM for unattended information. The first hypothesis we wanted to address was the *distance-related degradation of the quality* of the unattended stimuli. Specifically, if attentional resources spill over to unattended locations, then the quality of the unattended representations would gradually decrease with an increasing cue-probe distance (Downing and Pinker, 1985; Schmidt et al., 2002). For this purpose, we systematically varied the distance between retro-cued and probed locations in those trials which enabled us to study the changes in representational quality as a function of *cue-probe distance*.

The second hypothesis we wanted to address was the *distance-related degradation in the item-to-location bindings* of the unattended items. Specifically, we tested whether the strength of the binding of the unattended stimuli to their location is spatially graded as a function of the *non-target distance*. Based on the hypothesis that spatial imprecisions are higher for unattended representations in the vicinity of attended representations, binding errors are expected to be spatially graded with neighboring non-targets being more prone to be confusion errors than further away. We fitted an adapted version of the mixture model originally proposed by Bays et al. (2009) to the produced colors. The original model decomposed the recall performance into target responses, binding errors and guesses. Crucial to the purpose of the current study, we wanted to further trace the sources of binding errors in terms of their spatial origin relative to the focus of attention. Accordingly, we further decomposed the binding errors with respect to the distance between target and non-target locations. Moreover, our design enabled us also to study whether these binding errors–graded or not- were modulated by attentional cueing. Specifically, the *cue-probe distance* manipulation we administered to study the graded quality changes also allowed us to test whether shifting attention to unattended locations at a close distance from the attended location (e.g., cue and probe distance 1) results in more binding errors than shifting further away (e.g., cue and probe distance 2).

## Methods

### Participants

Twenty-one Ghent University students (4 males, 18–30 years, *M* = 23) participated for course credits. One participant was excluded as he did not finish the second session of the experiment. All participants had normal or corrected-to-normal vision and reported having normal color vision. The research complied with the guidelines of the Independent Ethics Committee of the Department of Psychology and Educational Sciences of Ghent University. All participants gave written informed consent.

### Task and Design

**Figure 1:**
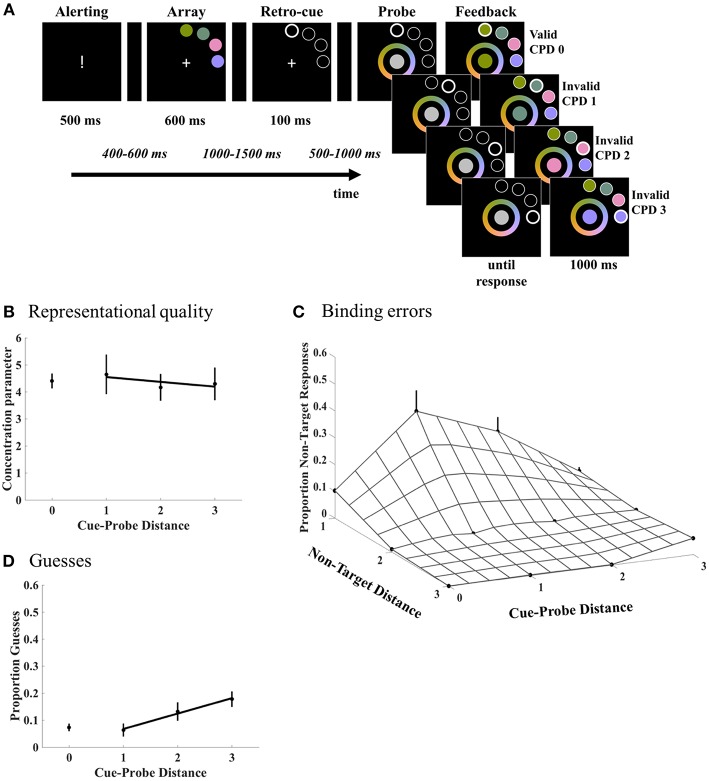
**(A)** Schematic overview of the continuous production task. Participants memorized a stimulus array of colored discs in order to precisely recall the color of the target at the probed location. A retro-cue guiding the focus of attention to the probed location (80% valid) was presented during the retention interval. Notice that in the invalid condition, the distance between the retro-cued and probed locations was equiprobably varied with increasing distance (CPD 1, 2, 3). Note that the noise patch in the inner surface of the color wheel presented during probe display is shown here in gray. In the actual experiment, this noise patch is randomly covered with all colors of the color wheel. **(B–D)** Mixture model components plotted as a function of Cue-Probe Distance. **(B)** The quality of the WM representations, as expressed by the concentration parameter κ, did not change as a function of the distance of the attentional shifts. **(C)** Binding errors significantly decreased with increasing cue-probe distance. Crucially, these binding errors defined as responses made according to non-targets were graded; non-targets close to the target location were confused as targets more often than remote non-targets. The gridded surface represents these binding errors varying as a function of both the non-target and cue-probe distance. **(D)** Random responses (guesses) also increased with increasing distance reflecting participants' failure to recall the memory information. The error bars denote the standard errors of the mean. Notice that the target responses representing correct item-location bindings are not plotted as the proportion target responses was obtained as a complement of binding errors and guessing.

A continuous color production task was administered that required selection of a color from a continuous color wheel. Participants viewed a stimulus array composed of four colored discs that were placed on an imaginary circle. They had to memorize the discs in order to report the color of a probe according to its location in the stimulus array (Figure 1A). Each disc location was probed (i.e., target) equally often with 25% probability. The probe (i.e., target) appeared at the retrospectively cued location in 80% of the trials (valid cue). Hence, in 20% of the trials the probe appeared on a different location than the retrospectively cued location (invalid cue). Crucially, the distance between cue and probe was controlled for the invalid trials where each cue-probe distance (CPD 1, 2, and 3) was balanced with 33% probability. Note that validly cued trials correspond to CPD 0. Finally, stimuli were either presented unilaterally in the upper left or upper right quadrant with 50% probability.

### Stimuli and Procedure

A white exclamation mark (!) announcing a new trial was presented against a black background in the middle of the screen for 500 ms. This was followed by a random interval ranging from 400 to 600 ms. The stimulus array of four discs appeared then for 600 ms. The discs (radius 0.5°) were placed on an imaginary circle at 5° eccentricity where the difference between the edges of each neighboring disc was 0.90°. These parameters exceed critical spacing measures (one tenth of the eccentricity), thus excluding the possibility that any distance effect in WM might be due to crowding effects during encoding rather than misbinding in WM (Levi, 2008; Emrich and Ferber, 2012). Each disc color was randomly chosen from a color wheel comprising of 360 color values with a minimal angular separation of 30°. The reason for employing a minimal angular separation was to make sure the colors were visually separable (Kiyonaga and Egner, 2016). The colors on the color wheel were evenly distributed along a circle on the CIE L^*^a^*^b color space centered at *L* = 50 with a radius of 22 on the surface of the a^*^b axes. All colors had an equal luminance and brightness and only varied in hue. A retro-cue was then presented 1,000–1,500 ms after the stimulus array for 100 ms. The retro-cue was presented as a thickening of one of the four placeholders of an empty array in which only the circumference of the disc was highlighted in white. A white fixation cross was presented during the retention interval between the stimulus array and the retro-cue.

After another random interval ranging from 500 to 1,000 ms with a white fixation cross, one of the four placeholder locations was randomly probed and remained on the screen until response without any response deadline. The probe display contained a color wheel with all 360 colors arranged on an annulus with inner radius of 0.46° and outer radius of 0.69°. A noise patch with a radius of 0.34° was presented within the color wheel in the center of the screen. The patch was randomly filled up with all colors of the color wheel. This patch changed into the selected color once participants responded. Participants responded by moving the mouse cursor on the color wheel and clicking on the left mouse button to indicate the color of the probed location. Responses were registered once the space bar was pressed which was immediately followed by feedback for 1,000 ms. Feedback consisted of filling up the discs with the colors as initially presented at the start of the trial, allowing the participant to verify his/her response. An intertrial interval of 750–1,250 ms was administered.

The experiment was run on two consecutive days with each session comprising 390 trials (15 blocks of 26 trials). Each session lasted about 1 h. Prior to the actual task, a practice block was administered to make the participants acquainted with the experiment. Participants were informed about the dependency between the cue, stimulus, and probe arrays. The task was programmed in Python, using the PyGaze toolbox (Dalmaijer et al., 2014) and PsychoPy (Peirce, 2007).

### Data Analysis

The analysis was performed by means of an adapted version of the methods used by Bays et al. (2009) (http://paulbays.com/). A continuous measure of the overall recall error was obtained on each trial as an angular deviation between the color reported by the participant and the true color of the target at the probed location. These values were then averaged separately for each cue-probe distance (CPD 0, 1, 2, and 3). To determine whether our manipulations for attentional cueing in WM were successful, validly cued trials (CPD 0) were compared to the invalidly cued trials (collapsed over CPD 1, 2, and 3) by a two-tailed paired *t*-test reflecting the cue validity effects. Crucially, these subject-averaged measures of recall error at varying cue-probe distances were then subjected to a regression analysis testing for the linear effects of CPD (1, 2, 3).

Next, to estimate the contributions of representational quality and of misbinding as possible consequences of an item not being selected for attentional processing, we applied an adapted version of the mixture model proposed by Bays et al. (2009). This mixture model decomposed three sources of errors: Gaussian variability around the target color, binding errors and a fixed probability of random guessing. Crucially, binding errors were previously calculated as a weighted sum of each non-target response (e.g., in Bays et al., 2009), yet, we here further decompose the binding errors as a function of spatial distance relative to probed location. The resulting model can be described as follow:

$$\begin{array}{l} p\left(\hat{\theta }\right)=\left(1-\gamma -\overset{m}{\underset{i}{\sum }}\beta_{i}\right)\phi_{\kappa }\left(\hat{\theta }-\theta \right)\\ \;+\;\gamma \frac{1}{2\pi }+\overset{m}{\underset{i}{\sum }}\beta_{i}\phi_{\kappa }\left(\hat{\theta }-\theta_{i}^{{\;}^{*}}\right), \end{array}$$

where θ is the true target color (probed), $\hat{\theta }$ the color reported by the participants, and ϕ_κ_ the von Mises distribution (circular analog of the Gaussian) with mean zero and concentration parameter κ. This parameter reflects the representational quality of WM items. More precisely, the concentration parameter κ corresponds to the variability in the target (and non-target) responses, with higher κ values indicating lower variability in responses, hence reflecting a higher representational quality. The probability of misbinding the target color with each of the non-target colors $\theta_{1}^{*},\theta_{2}^{*},\ldots ,\theta_{m}^{*}$ as function of their spatial distance to the target location is given by β_*i*_ with *i* = {1,2, …, *m*}, where *m* is the number of non-targets (in our experiment, *m* = 3). In other words, the misbinding components reflect erroneous item-to-location bindings taking into account the spatial distance between the non-target to the target locations. These misbinding components reflect the probability of each non-target at varying spatial distances from the target locations being erroneously reported as non-targets.

The probability of random guessing is captured by γ, reflecting participants' behavior when they did not remember the color of the probed location. The probability of target responses is obtained as the complement of guessing and misbinding, reflecting the correct item-to-location bindings. The maximum likelihood estimates of the parameters κ, β_1_, β_2_, β_3_, γ were separately obtained for each subject and cue-probe distance (CPD: 0, 1, 2, 3).

To determine whether our manipulations for attentional cueing in WM were successful, cue validity effects were tested for each obtained measure by two-tailed paired *t*-tests at an alpha level of 0.05. Crucial to the purpose of this study, subject-averaged measures of the concentration parameter κ at different cue-probe distances were then subjected to a regression analysis testing for the linear effects of CPD (1, 2, 3). This analysis was performed in order to quantify the modulations of quality of representations at uncued locations outside the focus of attention. Similarly, the subject-averaged probability of target responses and guesses obtained from the mixture model were separately subjected to the same analysis for the same purpose.

To test whether binding errors are spatially graded, non-target distance effect was first examined between valid (i.e., CPD 0) and invalid cue conditions collapsed over all cue-probe distances (i.e., CPD 1, 2, 3). A Repeated Measures ANOVA with cue validity (valid and invalid cues) and non-target distance (1–3) as within-subject factors was used to test this. The misbinding components of our model reflect the proportion of recall errors driven by the non-targets at different locations from the target location. Thus, the probabilities of responding with non-targets at each distance (non-target distance) that were derived from the model were entered into the analyses. Importantly with respect to the purpose of the current experiment, regression analyses were then performed on the linear effect of these misbinding errors as a function of non-target distance. Thus, the same probabilities of responding with non-targets at each distance (non-target distance) that were entered into the Repeated Measures ANOVA were entered into the regression analysis. The purpose of this analysis was to test whether these binding errors are graded as function of their distance to the target location or not. In a subsequent analysis, the non-target distance effect was studied at each cue-probe distance separately to examine whether the graded binding errors would be further modulated by the distance of the shifts of attention from the cued to the probed location. Differences in slopes between cue conditions were tested by one-tailed *t*-tests as our hypotheses were informed by previous findings (Rerko and Oberauer, 2013; Rerko et al., 2014; Sahan et al., 2015).

Important to note is that the mixture model that we described is an extension to that of Bays et al. (2009) and it therefore includes more free parameters. However, this requires caution in that more free parameters potentially increase the uncertainty of the model. This necessitates a quantification of the fit of our model in comparison to models with fewer parameters. A first model to which we compared our model is that of Bays et al. (2009) which included the concentration parameter (κ), the probabiliy of guessing (γ), and a compound probability for misbinding (β, disregarding the non-target distance). A second model to which we compared our model to was that of Zhang and Luck (2008) which did not have any misbinding component at all. For statistical comparison, we calculated the Bayesian information criterion (BIC) that provides us with a measure of relative strength in support of the model that also takes into account its complexity, here in terms of parameter uncertainty (Schwarz, 1978; Kass and Raftery, 1995). Following the model selection procedure outlined by (Lewandowsky and Farrell, 2010), we obtained BIC values for each subject's model fit to their data in the validly cued trials and averaged these BIC values (across subjects). The averaged BIC value was then compared across models. The model with the smallest BIC has been described as being the model with the highest posterior probability given the data, thus indicating the winning model (Lewandowsky and Farrell, 2010).

## Results

The overall recall error for validly cued trials at cue-probe distance 0 (*M* = 54.14°, *SE* = 12.42°) was significantly lower than for the invalidly cued (*M* = 70.33°, *SE* = 16.13°) trials collapsed over all other cue-probe distances [*t*_(19)_ = 5.60, *p* < 0.001, 95% *CI* = (10.14, 22.23)], suggesting that the attentional cueing in WM was successful. To determine whether recall errors in the invalidly cued trials differ over the relative distance between the cue and probe, recall errors were regressed to the cue-probe distance. The average recall errors at the cue-probe distances 1, 2, and 3 were 68.59° (*SE* = 4.34°), 68.54° (*SE* = 4.31°), and 73.87° (*SE* = 4.18°), respectively. The regression analysis did not reveal any modulation in recall error by cue-probe distance [β = 2.64, *SE* = 7.84; *t*_(19)_ = 1.51, *p* = 0.93, 95% *CI* = (−1.03, 6.31)].

In order to further investigate the sources underlying these overall recall errors, our model was applied to the data. We quantified the quality of WM representations, binding errors, guesses, and target responses at each cue-probe distance. Crucially, the binding errors presented here further traced the contribution of each non-target item at varying distances from the probed location. Figure 1 shows how these various components contributed to the overall recall errors. Regarding the first hypothesis of the distance-related degradation of the representational quality, there was no difference between valid and invalid conditions collapsed across CPD [*t*_(19)_ = 0.074, *p* = 0.94, 95% *CI* = (−0.90, 0.96)]. Contrary to our predictions based on the attentional gradient, neither was there a distance effect across the cue-probe distances 1, 2, and 3 (β = −0.08, *SE* = 0.18; *t*_(19)_ = −0.45, *p* = 0.66, 95% *CI* = (−0.39, 0.23)]. These results suggest that the representations in WM are maintained with an equal representational quality regardless of the cue-probe distance (Figure 1B).

As to our second hypothesis regarding the distance-related degradation in the item-to-location bindings, binding errors were modulated by the retro-cueing condition (Figure 1C). More precisely, the analysis revealed a main effect of cue validity [*F*_(1, 19)_ = 9.06, *p* = 0.007, $\eta_{p}^{2}$ = 0.32] with overall more binding errors in the invalid trials collapsed over all cue-probe distances (CPD 1, 2, and 3). Furthermore, a main of effect of non-target distance [*F*_(2, 18)_ = 29.31, *p* < 0.001, $\eta_{p}^{2}$ = 0.77] was obtained. Finally, the non-target distance by cue validity interaction was also significant [*F*_(2, 18)_ = 4.81, *p* = 0.021, $\eta_{p}^{2}$ = 0.35]. To quantify the effect of non-target distance in the overall recall errors, binding errors were regressed to the non-target distances. Regression analyses revealed that binding errors decreased with distance both in valid (β = −5.12, *SE* = 0.44; *t*_(19)_ = −11.403, *p* < 0.001, 95% *CI* = (−6.06, −4.18)] and invalid trials (β = −11.77, *SE* = 2.44; *t*_(19)_ = −4.82, *p* < 0.001, 95% *CI* = (−16.88, −6.66)]. The linear decrease in the invalid condition was significantly larger compared to the valid condition [*t*_(19)_ = 2.77, *p* = 0.012, 95% *CI* = (1.62, 11.67)].

In order to test the hypothesis whether the graded binding errors are further modulated with shifts in mental space across varying cue-probe distances, we tested for a linear effect of the non-target distance to binding errors at each cue-probe distance separately. At CPD 1, the linear effect of misbinding non-targets with distance was significant [β = −17.85, *SE* = 3.79; *t*_(19)_ = −4.72, *p* < 0.001, 95% *CI* = (−24.40, −11.30)]. Similarly, the linear effect at CPD 2 was also significant [β = −12.14, *SE* = 2.60; *t*_(19)_ = −4.67, *p* < 0.001, 95% *CI* = (−16.64, −7.64)]. However, the distance effect was absent at CPD 3 [β = 0.15, *SE* = 1.60; *t*_(19)_ = 0.095, *p* = 0.925, 95% *CI* = (−2.62, 2.93)]. These slopes were also subjected to a regression analysis to test our second hypothesis that the graded shape of misbinding would be more profound with small shifts compared to larger shifts of attention. Regressing the slopes across cue-probe distances revealed a linear decrease in the steepness of the slopes with increasing cue-probe distance [β = −9, *SE* = 2.1; *t*_(19)_ = −4.29, *p* < 0.001, 95% *CI* = (−5.37, −12.63)].

The analyses performed on the probability of guesses showed that participants made significantly fewer guesses on the validly cued trials compared to the invalidly cued trials collapsed over all cue-probe distances [*t*_(19)_ = −2.50, *p* = 0.022, 95% *CI* = (−9.40, −0.81)]. To determine whether guesses in the invalidly cued trials differ over the relative distance between the cue and probe, the proportion of guesses was examined as a function of cue-probe distance (Figure 1D). The regression analysis revealed a linear increase in proportion of guesses with increasing cue-probe distance [β = 5.70, *SE* = 1.76; *t*_(19)_ = 3.23, *p* = 0.004, 95% *CI* = (2.65,8.74)].

The probability of the target responses reflecting correct item-to-location binding complemented the binding errors. Target responses were modulated by the retro-cueing condition in that, the probability of target responses for validly cued trials (*M* = 0.81, *SE* = 0.02) was significantly higher than for the invalidly cued trials collapsed over all cue-probe distances [*M* = 0.60, *SE* = 0.03; *t*_(19)_ = 3.95, *p* = 0.001, 95% *CI* = (8.01, 26.13)]. To determine whether target responses in the invalidly cued trials vary with distance between the cue and probe, the proportion of target responses was examined as a function of cue-probe distance. The average proportion of target responses at the cue-probe distances 1, 2, and 3 were 0.54 (*SE* = 0.07), 0.59 (*SE* = 0.05), and 0.67 (*SE* = 0.03), respectively. The regression analysis revealed a linear increase in proportion of target responses with increasing cue-probe distance [β = 6.04, *SE* = 3; *t*_(19)_ = 2.01, *p* = 0.03, 95% *CI* = (0.85, 11.24)]. The increase in proportion target responses indicates that recall performance to probes became better at larger distances from the initially retro-cued locations.

As a validation step, we compared the group level BIC value of our model (*BIC* = 1419) to the model with a compound misbinding component (*BIC* = 1458) and the model without the misbinding component (*BIC* = 1460). The BIC values for all subjects except one were smaller for our model compared to the other two models. These results suggest that our model provides a comparable, if not better fit to the data relative to the established models in literature with fewer parameters supporting the validity of our approach.

## Discussion

The purpose of Experiment 1 was to investigate the fate of unattended representations in WM. We addressed how uncued memory information is influenced by the attentional prioritization of cued information. Previous research suggested that there is an attentional gradient around the cued location making it harder to reject these neighboring non-targets (Sahan et al., 2015) and more prone to be erroneously recalled as targets compared to more remote non-targets (Rerko et al., 2014). However, it is not clear how these graded distance effects emerge in WM. Two hypotheses were tested in the current study. One stated that the quality of the unattended items itself is spatially graded in WM. The quality of the uncued representations did not show any modulation by the spatial distance to the cued location. This suggests that the attentional resources are equally distributed across the uncued representations and that the quality of these uncued representations is not subjected to an attentional gradient.

The second hypothesis is that unattended items in the vicinity of the attended items are more vulnerable to spatial confusions due to weakened item-to-location bindings, a vulnerability that gradually decreases with increasing spatial distance (Bays and Husain, 2008; Oberauer and Lin, 2017). In line with this prediction, binding errors were indeed spatially graded. Specifically, non-target colors near the probed location were more prone to be bound to the wrong location, namely that of the target compared to more remote non-targets. Moreover, this gradient in binding errors varied with the distance of the attentional shifts: the smaller the shift the larger the graded intrusions of non-target colors. These graded binding errors were further complemented with the finding that proportion of target responses, corresponding to accurate color-location bindings were compromised with distance of the shifts. More precisely, the bindings of uncued color representations to their respective locations were less accurate near the cued location compared to uncued colors further away. Moreover, if participants failed to remember the colors at larger cue-probe distances they responded randomly. In conclusion, the combined effect of spatial distance on the correct and incorrect color-location bindings suggests that the fate of unattended representations is graded in terms of the spatial imprecision in the locations between the targets and non-targets in WM rather than being imprecise in feature space (i.e., color).

## Experiment 2

In order to confirm the graded nature of binding errors of Experiment 1, we conducted a categorical version of the production task. More precisely, the task was to recall the exact color at a probed location from a visual array held in WM. The stimuli in the visual array were now limited to a distinct set of five categorical colors (i.e., red, green, blue, yellow, and pink as clearly distinguished colors) hence forcing participants to choose among five response alternatives respective to the colors. The advantage of this approach is that the reported responses can no longer deviate from the true color on the color space. This way, we can directly assess the graded nature of binding errors in terms of the spatial imprecision of the target and non-target locations in WM rather than derive it in a model-based way from the imprecision in color space as obtained from the continuous production task. We predicted that non-targets near the focus of attention would be gradually more prone to misbinding compared to non-targets at further distances. Furthermore, the findings of Experiment 1 suggest that this pattern of graded binding errors would be more pronounced when target locations are invalidly cued.

## Methods

### Participants

Twenty Ghent University students (2 males, 17–28 years, *M* = 18.5, none participated in Experiment 1) participated in return for financial compensation. All participants had normal or corrected-to-normal vision and reported having normal color vision. The research complied with the guidelines of the Independent Ethics Committee of the Department of Psychology and Educational Sciences of Ghent University. All participants gave written informed consent.

### Task and Design

The task and design are identical to Experiment 1 with the following exceptions. A categorical production task was administered where the stimulus set was limited to categorically distinct colors. The set size was increased to five as the categorical version of the task is less demanding. This allowed us to study the distribution of non-target colors misremembered as a function of their spatial distance to the probed locations (non-target distances 1, 2, 3, 4). Each disc location was equally often probed with 20% probability. The probe (i.e., target) appeared at the retrospectively cued location in 80% of the trials (valid cue). Hence, in 20% of the trials the probe appeared on a different location than the retrospectively cued location (invalid cue). Contrary to Experiment 1, the relative distance between the cued and probes was not systematically controlled as the main aim here was to replicate the general finding of graded bindings errors.

### Stimuli and Procedure

**Figure 2:**
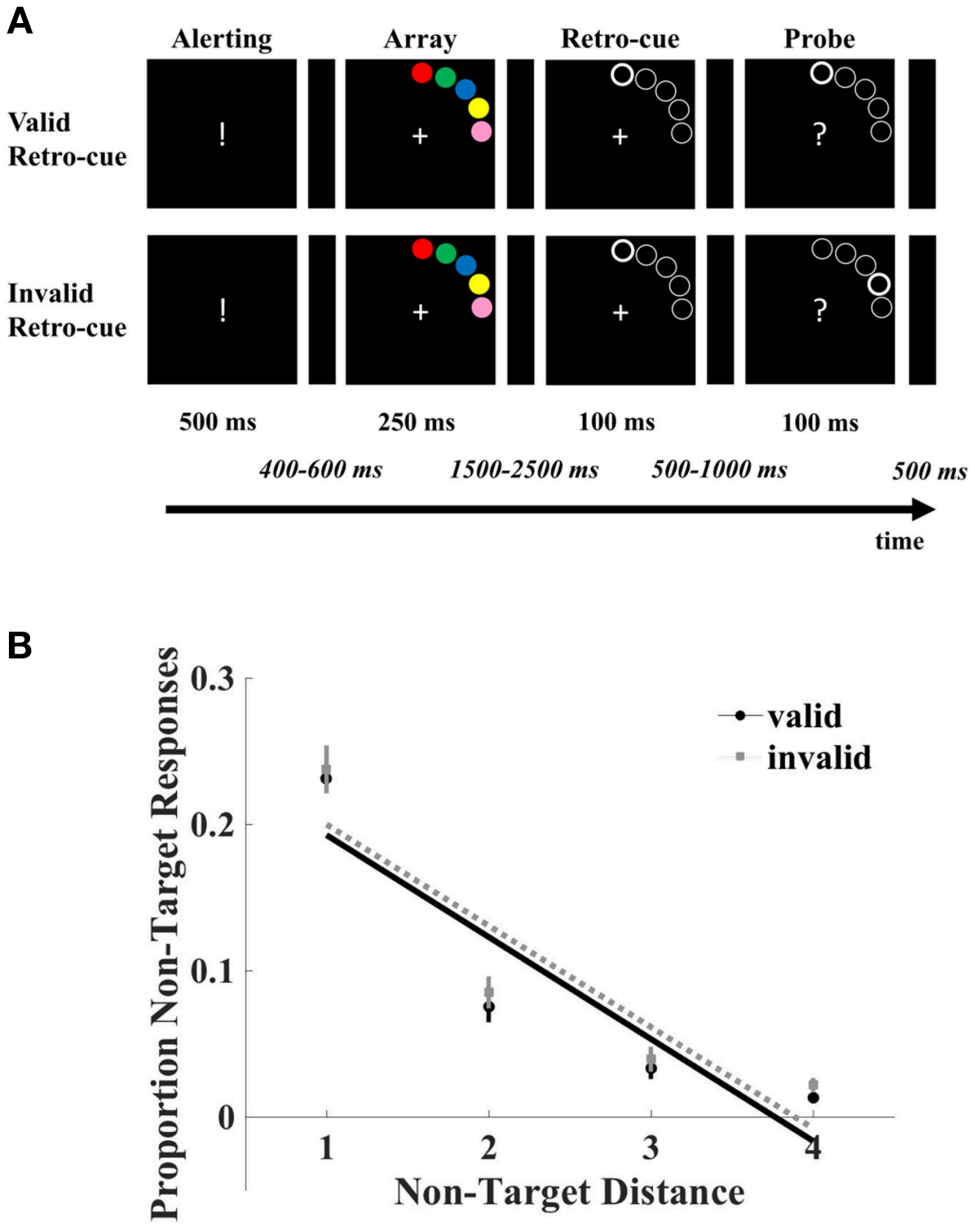
**(A)** A schematic overview of the categorical delayed production task. Participants memorized a stimulus array of colored discs in order to recall the color of the target at the probed location. A retro-cue guiding the focus to the probed location (80% valid) was presented during the retention interval. **(B)** The proportion of errors in recall plotted as a function of the distance between the non-targets and probed target both in the validly and invalidly cued trials. Recall in errors were all significantly modulated by the distance of non-target colors. The error bars denote the standard errors of the mean.

Parameters were the same as in Experiment 1 with the following exceptions (Figure 2A). The stimulus array consisted of five discs that appeared for 250 ms. The five discs (radius 0.5°) were placed on an imaginary circle at 8° eccentricity where the difference between the edges of each neighboring disc was 1.50°. The colors red, green, blue, yellow, and pink (with the CIE L^*^a^*^b color space values *L* = 53, *a* = 80, *b* = 67; *L* = 88, *a* = −86, *b* = 83; *L* = 32, *a* = 79, *b* = −107; *L* = 97, *a* = −21, *b* = −94; *L* = 81, *a* = 28, *b* = 5, respectively) were randomly assigned to the discs. Along with the probe, a question mark was presented that indicated the participants to select the color of the probed location from among the colors of the stimulus array. Responses were registered through a keyboard with a fixed response mapping to the colors that participants had to learn prior to the experiment (F, G, H, J, and K respectively for red, green, blue, yellow and pink). No feedback was given in this experiment. An intertrial interval of 500 ms was administered. The experiment was comprised of 400 trials (16 blocks of 25 trials) and lasted for about 1 h. Prior to the actual task, a practice block was administered to acquaint the participants to the experiment. Participants were informed about the dependency between the cue, stimulus and probe arrays. The task was programmed using an updated version of the experiment programming library TScope5 in C/C++ (Stevens et al., 2006).

### Data Analysis

The mean proportion of errors committed in the validly and invalidly cued trials were analyzed as a function of their distance to the probed location using Repeated Measures ANOVA with cue validity (valid and valid cues) and non-target distance (1–4) as within-subject factors. Multivariate test results for repeated measures are reported. To quantify the distance effect, regression analyses were performed to test for linear effects (following Lorch and Myers, 1990). Differences in slopes between cue validity conditions were tested by one-tailed *t*-tests as our hypotheses were informed by previous findings (Sahan et al., 2015).

## Results

The analyses revealed a main effect of cue validity [*F*_(1, 19)_ = 7.43, *p* = 0.013, $\eta_{p}^{2}$ = 0.28] with a higher proportion of errors in the invalid trials (*M* = 0.38*, SE* = 0.029) compared to valid trials (*M* = 0.35*, SE* = 0.026). Furthermore, a main of effect of non-target distance [*F*_(3, 17)_ = 109.32, *p* < 0.001, $\eta_{p}^{2}$ = 0.95] was obtained. The non-target distance by cue validity interaction did not reach significance (*F* < 1). Crucially, regression analyses revealed that errors decreased with distance both in valid [β = −6.97, *SE* = 0.31; *t*_(19)_ = −22.63, *p* < 0.001, 95% *CI* = (−7.50, −6.44)] and invalid trials (β = −6.93, *SE* = 0.19; *t*_(19)_ = −14.17, *p* < 0.001, 95% *CI* = (−7.77, −6.08)]. As indicated by the absence of an interaction between cue validity and non-target distance, the slopes did not differ [*t*_(19)_ = −0.12, *p* = 0.90, 95% *CI* = (−0.82, 0.72)]. See Figure 2B.

## Discussion

The main goal of this experiment was to study binding errors by means of a model-free approach in validation of the graded binding error findings of Experiment 1. For this purpose, we asked participants to report the target color from among categorically distinguishable non-target colors rather than (model-based) deriving the binding errors from the continuous reports as in Experiment 1. This allowed us to descriptively chart down the spatial error patterns in binding non-target colors to target locations without modeling. Overall, the results of this experiment replicate the findings of Experiment 1 with a model-free approach (Rerko and Oberauer, 2013; Rerko et al., 2014; Sahan et al., 2015). More errors were made in the invalidly cued trials compared to the validly cued trials. The nature of the errors suggests that there is a systematic bias toward non-target colors near the attended locations compared to non-targets that are further away in both validly and invalidly cued conditions with overall more errors in the invalidly cued condition. Our results provide new insights into how representations outside the focus attention are maintained when the spatial distance of non-target items to the target items is taken into account (Bays et al., 2009; Oberauer and Lin, 2017). Namely, non-target representations are spatially graded in terms of their likelihood to be confused with the target representations. In line with previous research (Rerko and Oberauer, 2013; Rerko et al., 2014; Sahan et al., 2015) we empirically showed that binding errors are graded even when there is no need to retain the individual items with high precision.

## General Discussion

The present study investigated the fate of WM representations outside the attentional focus. Work with the retro-cueing paradigm was so far primarily focused on the prioritization of the cued representation at the expense of uncued representations (Gazzaley and Nobre, 2012; Ma et al., 2014). However, what is unclear is how the cost of this prioritization is divided across the unattended representations in WM. In principle, there are two possible consequences that attentional selection could have on the unattended representations in WM. A first hypothesis states that the quality of the uncued items is spatially graded in WM and that this affects the salience of these distractors (Downing and Pinker, 1985; Schmidt et al., 2002). A second hypothesis states that the item-to-location bindings of the uncued representations are weakened in WM making the unattended representations near attended locations more vulnerable to binding errors (Bays et al., 2009; Oberauer and Lin, 2017). In support of the second hypothesis, we found that the binding errors were spatially graded while the representational quality of unattended items was not spatially graded. Specifically, the vulnerability of non-targets being misbound gradually decreased with the spatial distance to the target location. These spatially graded binding errors were even more pronounced when the focus of attention was shifted from the retro-cued to the probed location.

A first implication of our results is that the discrepancy between the binding errors being spatially graded and quality of WM representations not being spatially graded suggests that the representational quality of the unattended memory items is distinct from how successfully those representations are bound to their distinct spatial locations. The observed distance effects pertain to spatial imprecision of the item-to-location bindings of the unattended representations rather than affecting the quality of the WM contents at unattended locations themselves. One potential explanation to account for this finding is that higher-order information can be abstracted from the precise continuous visual information and retained as a conceptual gist of the representations (e.g., Lampinen et al., 2001; Hollingworth and Henderson, 2002; Oliva, 2005). According to this view, it is likely that the continuous information of the WM items is maintained as conceptual approximative entities (e.g., “reddish,” “greenish,” etc.) that are abstracted away from the sensory levels of representation. Our findings suggest that these abstracted higher-order representations are stored with an equal degree of representational quality regardless their spatial distance to the attended locations. To illustrate this idea more clearly, an unattended representation with the abstract label “reddish” at a closer spatial distance to the attended location is not phenomenologically richer, because of an attentional gradient as we predicted, than an unattended representation of “greenish” at a larger spatial distance. The attentional gradient does thus not affect this abstract feature of the representations. Rather, it has an impact on the binding strengths of these representations to their specific locations. This explanation that representational gists might be extracted remains speculative and additional research is needed to see how much credence can be given to this account.

Although we did not find any support for the hypothesis that there is an attentional spillover to unattended locations in terms of the representational quality (Downing and Pinker, 1985; Schmidt et al., 2002), Souza et al. (2018) recently showed that in a change-detection paradigm items at probed locations close to the initially pre- or retro-cued locations were recognized better than items further away. Specifically, probe recognition performance gradually dropped with increasing spatial distance between the probed and cued locations supporting the attentional spillover hypothesis. One potential reason for this inconsistency between the findings of Souza et al. (2018) and ours is the difference between recognition and recall processes. Another methodological consideration pertains to the validity of the retro-cues in that it can determine the degree with which uncued representations remain accessible in WM. For instance, Gunseli et al. (2015) showed that in a continuous orientation production task, uncued representations were recalled with a higher precision when retro-cues were less reliable (i.e., 50% validity) compared to when retro-cues had a higher reliability (i.e., 80% validity). This suggests that when uncued representations have a higher likelihood of being probed, it is more advantageous to keep those representations active in WM. Based on this consideration, it is likely that Souza et al. (2018) found the attentional gradient because they employed retro-cues with a relatively lower reliability (i.e., cue-validity 60%, experiment 2) compared to our study (i.e., cue-validity 80%). This distinction suggests that despite the fact that unattended representations in our experiment are less accessible in terms of their representational quality, neighboring probes were nevertheless more prone to be confused as targets. A matter for future research is to determine whether the attentional spillover arises with less reliable retro-cues. However, our findings point to an interesting conclusion that spatially graded effects appear at the level of item-to-location bindings but not at the level of the quality of WM representations.

Previous research has shown that the representational quality is subjected to cue-validity effects with higher representational quality for the validly cued compared to invalidly cued conditions while this overall cue-validity effect was absent in our data (e.g., Williams et al., 2013; Gunseli et al., 2015; van Moorselaar et al., 2015). What drives the difference in the retro-cue effects between these studies and ours remains unclear. However, one potential reason for this discrepancy is that the methodological differences across studies could have led participants to adopt different strategies in response to the retro-cues. For instance, Gunseli et al. (2015) pointed out that retro-cue effects are affected by their reliability. Specifically, at an 80% validity compared to a 50% validity, invalidly cued items were not only less likely to be recalled, but also recalled with a lower representational quality. Gunseli et al. (2015) argued that this reflects a strategic adaptation of participants to the reliability of cues in their environment. Although there are obvious methodological differences between the studies (e.g., Williams et al., 2013; Gunseli et al., 2015; van Moorselaar et al., 2015), it is less obvious whether these differences could meaningfully interact with the representational quality of the recalled items. For example, the stimuli in Gunseli et al.'s (2015) work were spaced out much more widely than the stimuli in our experiments, as ours was specifically designed to answer a question about cue-target distance effects on recall. This likely impacted the recall probability of non-target items, which was at around 0.2 in Gunseli et al.'s (2015) study, but approached 0.4 for stimuli with a cue-probe distance of 1 in our study (and is only about 0.1 for cue-probe distances of 0 and 3). It could well be that aforementioned differences between the experimental environments of, for instance Gunseli et al.'s (2015) study and our own impacted participants' strategies in different ways. Future research is required to investigate what these strategic differences are exactly, and how stimulus features impact them.

Our observation that binding errors are spatially graded converge with studies outside the context of retro-cues where binding errors have also been found to be spatially graded in that, non-targets near the target locations were more likely to be confused than non-targets further away at any probed location (Emrich and Ferber, 2012; Bays, 2016; Pratte, 2018). This overlap may suggest that attentional prioritization is accomplished with comparable consequences to non-targets at different stages of WM processing; while retro-cues may induce a gradient during retention, probes that were not preceded by retro-cues may also induce a similar gradient at retrieval. The further modulation of the binding errors we observe is indicative of this graded effect at different stages. The graded binding errors were more pronounced when another location was probed than the initially retro-cued one, an effect that decreased with increasing cue-probe distance.

One potential limitation of the current study is that the priority status of some probe positions could be biased leading to strategic resource allocation policies favoring one position over the other. Klyszejko et al. (2014) showed that varying the probabilities with which certain items are tested monotonically affects memory precision to the degree of the testing probability. We controlled for the cue-probe distances (CPD 1-2-3) in a balanced design where each distance was presented with an equal probability (33%). However, the likelihood of testing the outer positions was in imbalance with the intermediate items as CPD 3 could only be tested at the outer locations. Hence, the combination of our stimulus configuration and the experimental design may have induced a bias in the priority maps. As suggested by Klyszejko et al. (2014), subjects could have strategically allocated more resources to the outer positions that in turn could explain that subjects made the least binding errors at the largest cue-probe distance. However, a closer inspection of the random guesses at those cue-probe distances argues against this possibility. The random guesses monotonically increased with the cue-probe distance while it is expected that random guesses should decrease in case the outer locations were strategically prioritized.

A second implication is that our findings point to the importance of considering the spatial distance between memory representations regardless of the theoretical accounts modeling binding errors, whether it be the resource (Bays and Husain, 2008) or interference accounts of WM (Oberauer and Lin, 2017). Within the framework of resource models, our findings suggest that there is graded allocation of attentional resources at the level of item-to-location bindings in WM that is underlying the increased confusions of non-targets near the attended compared to unattended locations further away. Within the framework of the interference models (Roggeman et al., 2010; Oberauer and Lin, 2017), competition between neighboring item-to-location bindings makes these items more prone to confusions. Thus, while we showed that the binding strengths are spatially graded in WM, the question how the attentional gradient is theoretically implemented is a matter for future research.

The modeling implications of our study is that the parameters characterizing the representational quality and binding strengths in Experiment 1 were obtained by modifying the mixture model originally developed by Bays et al. (2009). While the binding errors in the original mixture model were calculated as a weighted sum of each non-target response, our model decomposed the binding errors with reference to their spatial distance to the target location. Thereby, our findings provide an extension to the mixture model of WM by taking into account the distance of the non-target locations in WM (Bays et al., 2009). Important to note is that we used a low number of trials to fit the mixture model (*n* < 50). In principle, this could have impacted the reliability of our parameter estimates. However, the model-based findings of Experiment 1 were further validated by the model-free results in Experiment 2. These results confirmed the importance of considering the spatial distance of unattended representations in recalling from WM. Alternatively, our findings are also compatible with the interference model proposed by Oberauer and Lin (2017). In their proposal, the occurrence of binding errors was modeled by a binding component on which a graded weighting was applied depending on the distance of the non-targets. This entails that the binding errors were spatially constrained with the gradient parameter. In contrast, with our approach we aimed to reveal the graded nature of binding errors without putting such constraints through a parameter in the model. Therefore, the current study presents direct empirical evidence for the graded contribution of the non-target locations at increasing spatial distances to the target location.

One can argue that the graded strength of bindings arises due to competition between items already occurring at the level of encoding (Levi and Carney, 2009; Emrich and Ferber, 2012; Souza et al., 2018). For instance, Emrich and Ferber (2012) showed that increasing the visual competition between WM items by lowering the inter-item spacing resulted in increased binding errors. Similarly, Souza et al. (2018) showed that the spatially graded recognition performance of unattended items, specifically better performance at small cue-probe distances compared to larger cue-probe distances, only emerged when the actual physical spacing was also reduced. Although we cannot completely rule out the possibility that there is some competition at the level of encoding, it is difficult to explain our findings merely by visual crowding. More precisely, we did not find any spatial gradients pertaining to the quality of WM representations even though the inter-item spacing in our Experiment 1 was half the size (11.25°) of the inter-item spacing in Souza et al. (2018) (22.5°) at the exact same eccentricity (5°). Thus, under these circumstances one would expect some spillover due to visual crowding. Furthermore, we conducted a pilot experiment similar to our Experiment 2 in which we only employed pre-cues that guide attention to perceptual information prior to the appearance of the stimulus array to gate the information into WM at the level of encoding (Supplementary Material). We reasoned that if our graded binding errors are only due to the visual crowding, then we should observe a similar degree of spatially graded binding errors for both the internally and perceptually shifted attention. We found that the spatially graded recall errors (i.e., gradually more neighbor confusions than confusions with further away non-targets) were more pronounced in retro-cue compared to the pre-cue version of the task. This suggests that visual crowding is not sufficient to explain the degree with which the spatially graded binding errors occur and that it must take place at later stages of processing during maintenance or even later during retrieval.

Moreover, the graded binding strengths for unattended representations we observed may only hold when feature-location bindings are relevant to the task. It remains an open question whether these graded binding strengths also occur when there is no reference to space such as in sequentially encoded WM items. According to position marker models, sequentially encoded information is embedded in an internal coordinate system on which spatial attention operates similar to external spatial attention (Abrahamse et al., 2014). Although speculative, it is then possible that sequentially encoded information is spatially represented and retro-cueing could have a graded effect on the unattended representations similar to its effect on item-to-location bindings. Future research should explore to what extent our graded binding strengths are generalizable to sequentially encoded WM representations.

Lastly, although the main research question under investigation was whether binding accuracy or the representational quality was impaired with distance, the target responses and guesses that were obtained from the mixture model followed a complementary pattern to the binding errors. While binding errors overall dropped, correct target responses, and guesses both monotonically increased with cue-probe distance. These findings have implications for theories that stipulate how exactly attention is divided across the unattended locations. Contrary to the hypothesis of a graded distribution in WM (Rerko and Oberauer, 2013; Sahan et al., 2015), center-surround models postulate a non-monotonic distribution (Hopf et al., 2005; Störmer and Alvarez, 2014; Kiyonaga and Egner, 2016). There, it is stated that the enhanced processing advantage at cued locations turn into inhibition for the area surrounding the focus of attention. This inhibition then gradually diminishes with increasing distance eventually leading to a Mexican-hat like distribution. Our approach of decomposing performance into binding errors, target and non-target responses in Experiment 1 and laying those measures out in terms of the spatial distance of attentional shifts is particularly informative in resolving inconsistencies between these models. Guesses and target responses are inconsistent with the center-surround models as a suppressed non-target neighbor would be harder to recall which would have resulted in a higher proportion of guesses. Both the graded nature of binding errors and the pattern of guesses and target responses that monotonically varied as a function of distance in attentional shifts are consistent with the findings of an attentional gradient in WM (Rerko and Oberauer, 2013; Rerko et al., 2014; Sahan et al., 2015).

To conclude, we propose that spatial gradients do not apply at the level of stimulus representation but at the level of binding the stimulus to its position. WM items that are not attended have a similar degree of representational quality regardless of their distance to the attended locations. However, these unattended memory representations -that are stored with the same representational quality- are themselves prone to confusions due to spatial degradation in WM.

## Data Availability

The datasets generated for this study are available on request to the corresponding author.

## Author Contributions

MS designed research. MS and ED performed research and analyzed data. MS, ED, TV, MH, and WF wrote the paper.

### Conflict of Interest Statement

The authors declare that the research was conducted in the absence of any commercial or financial relationships that could be construed as a potential conflict of interest.
