# Supplementary material for: The Graded Fate of Unattended Stimulus Representations in Visuospatial Working Memory
Source: Front Psychol. 2019 Feb 26;10:374. doi: 10.3389/fpsyg.2019.00374 (PMC6399423; doi:10.3389/fpsyg.2019.00374)
Supplement: Supplementary file 1 [file Table_1.DOCX]

**Appendix**

Contrary to the argument that binding errors take place at the stages of maintenance or retrieval (Bays et al., 2009; Bays, Gorgoraptis et al., 2011; Gorgoraptis et al., 2011), it is likely that visual crowding during encoding induces competition among stimuli which then leads to the binding errors in WM (Emrich & Ferber, 2012). In order to rule out a potential effect of visual crowding, the same task as in Experiment 2 was conducted with pre-cues that prospectively guide attention to perceptual space and select the relevant targets among visual distractors. We hypothesized that if crowding effects underlie our distance effects in retro-cue trials as in Experiment 2, then a similar effect of distance is expected in pre-cue trials.

**Method**

**Participants.** Twenty other Ghent University students (2 males, 18-30 years, *M* = 18) than in Experiments 1 and 2 participated in return for financial compensation. All participants had normal or corrected-to-normal vision and reported having normal color vision. The research complied to the guidelines of the Independent Ethics Committee of the Department of Psychology and Educational Sciences of Ghent University. All participants gave written informed consent.

**Task, Design and Procedure*.***  The parameters were the same as in Experiment 2 except for time at which the cue was presented. A white exclamation mark (!) announcing a new trial was presented against a black background in the middle of the screen for 500 msec. This was followed by a random interval ranging from 400-600 msec. The pre-cue was then presented for 100 ms which then was followed by a bank delay that ranged between 1500-2500 msec. Subsequently, the stimulus array was presented for 100 msec. After another random interval ranging from 500-1000 msec, the probe was presented for 100 msec.

**Data Analysis.** As our main purpose was to rule out a potential visual crowding effect, we limited our analyses to a comparison of the distance effects among pre- and retro-cue conditions only for the validly cued trials. Repeated measures ANOVA was conducted for the distance of non-targets (Distance 1,2,3,4) as within-subjects and Cue Type (pre- and retro-cue) as between-subjects factor. Crucially, regression analyses were performed to quantify the difference in the linear distance effects between pre- and retro-cue conditions (following Lorch & Myers, 1990). Differences in slopes between cue types were tested by two-tailed t-tests as in Sahan et al. (2015). An alpha level of .05 was applied and Bonferroni correction was used on multiple tests to control for false-positives.

**Results and Discussion**

The analyses revealed a main effect of *Cue Type* [*F*(1,38)=100.59, *p<*.001, *η_p_^2^*=.73] with overall more errors in the retro-cue relative to the pre-cue condition. Furthermore, a main of effect of *Non-Target Distance* [*F*(3,36)=145.16, *p*<.001, *η_p_^2^*=.92] was obtained. The analysis also revealed a *Cue Type* by *Non-Target Distance* interaction [*F*(3,36)=75.19, *p*<.001, *η_p_^2^*=.86]. Crucially, regression analyses revealed that errors decreased with distance both in retro- (*β*=-6.97, *SE*=.31; *t*(19)=-22.63, p<.001, *95% CI*=[-7.50,-6.44]) and pre-cue conditions (*β*=-1.13, *SE*=.22; *t*(19)=-5.01, p<.001, *95% CI*=[-1.52,-.75]). The slopes were significantly steeper in the retro-cue condition relative to the pre-cue condition (*t*(38)=15.36, *p*<.001, *95%CI*=[5.06,6.61]). These findings suggest that visual crowding is not sufficient to explain the degree with which the spatially graded binding errors occur that must take place at later stages of processing during maintenance or even later during retrieval. See figure 1.


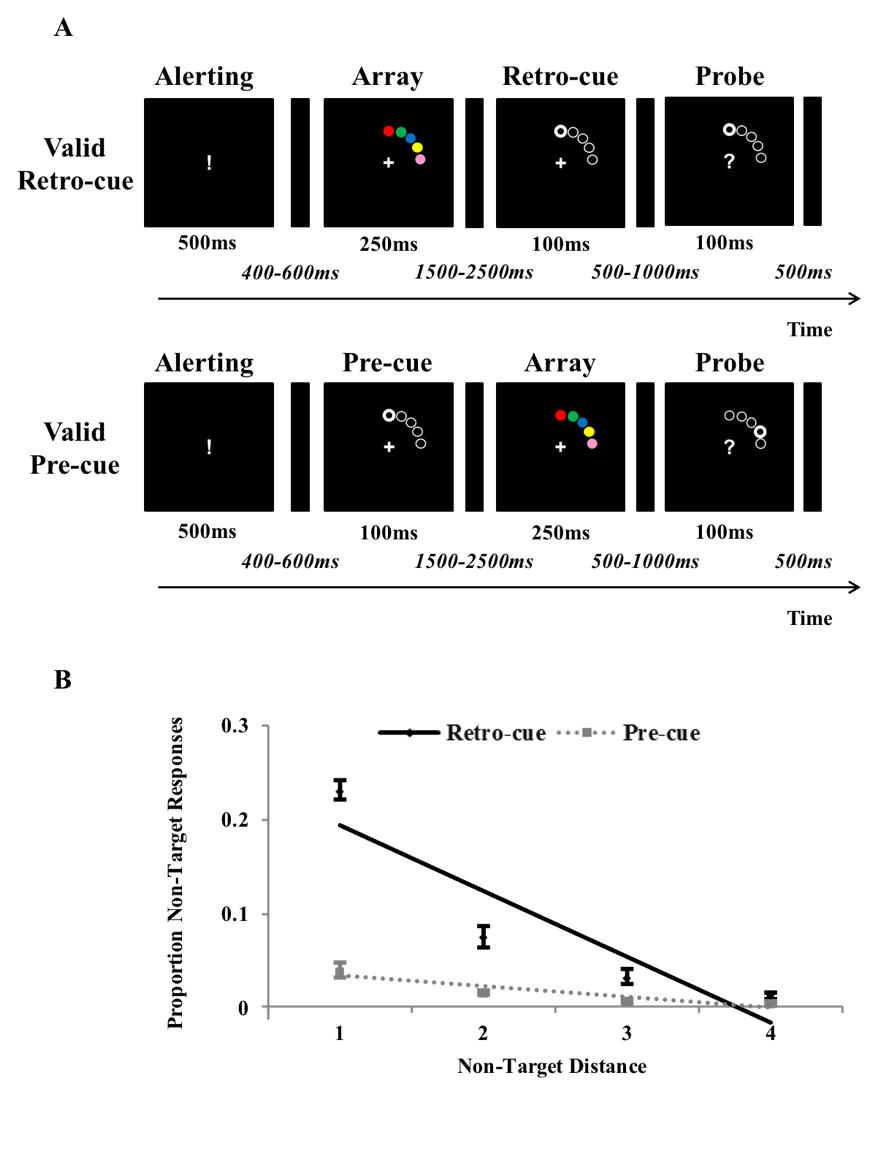


**Figure 1** A schematic overview of the categorical delayed production task (A). Participants memorized a stimulus array of colored discs in order to recall the color of the target at the probed location. In the retro-condition, cues retrospectively guided the focus of attention to the probed location (80% valid) during the retention interval. In the pre-cue condition, cues prospectively guided the focus of attention to the probed location (80% valid) prior to the presentation of the array. The proportion of errors in recall is plotted as a function of the distance between the non-targets and probed target both in the retro- and pre-cue trials (B). Notice that the distance effects plotted here are only considering the validly cued trials. Recall in errors were all significantly modulated by the distance of non-target colors. However, this effect was more pronounced in the retro-cue condition suggesting that it is not visual crowding but a decay in spatial resolution in WM that is underlying the results. The error bars show the within-subject standard errors of the means.
